# Comment on Laroche, E.; L’Espérance, S. Cancer Incidence and Mortality among Firefighters: An Overview of Epidemiologic Systematic Reviews. *Int. J. Environ. Res. Public Health* 2021, *18*, 2519

**DOI:** 10.3390/ijerph19137558

**Published:** 2022-06-21

**Authors:** Tee Guidotti

**Affiliations:** Occupational + Environmental Health & Medicine, c/o Advanced Technologies and Laboratories, Gaithersburg, MD 20878, USA; tee.guidotti@gmail.com

This is a letter to the Editor critiquing Laroche and L’Esperance, “Cancer Incidence and Mortality among Firefighters: An Overview of Epidemiologic Systematic Reviews” [1].

The literature on firefighters probably represents the second-largest body of data on any civilian occupation (after asbestos workers) and, therefore, plays an important role in occupational epidemiology in general.

On 3 March 2021, the *International Journal of Environmental Research and Public Health* published yet another review of cancer risk among firefighters. This particular study is distinguished by its apparent detail and air of authority, as well as its inclusion of grey literature. In fact, the paper perpetuates misconceptions about the firefighting literature that should have been laid to rest long ago.

The study perpetuates most of the failings of the recent literature on firefighting and occupational health. It treats firefighting as a single, immutable occupation although chemical exposures, firefighting practices, and health protection have changed dramatically in recent years.

The “methodology” of the paper is described as a “qualitative synthesis”. However, their described methodology of “systematic review” does not mean “structured review”, as the modifier “systematic” would normally imply, but rather their narrative summary of what they inferred from a superficial reading. The reading is superficial. The reading is superficial both because it failed to note observations in the original papers (such as the historical evolution of risk in firefighting) and because they excluded the original authors’ own detailed systematic and structured analyses presented in books and methodologically informed papers that were inappropriately excluded by their search strategy, as described below.

In practice, the study relies heavily on only six studies. It indiscriminately treats disease risk reported from 1990 the same as in recent studies and meta-analyses of overlapping cohorts as entirely independent studies. There is hardly any discussion of power (an important issue in these studies), the conspicuous absence of subgroup analysis in work assignments in this literature, and the limitations of the length of service as an imperfect exposure metric.

The study also continues a long and unaccountable tradition in the firefighting literature of ignoring inconvenient but obvious methodological limitations, such as the aggregation of biologically distinct diagnoses with known differences in etiology into aggregate categories (such as non-Hodgkin lymphoma) or overly broad organ-specific tumor sites that dilute disease risk by the inclusion of conditions unlikely to have an association with firefighting (e.g., the many types of brain cancer, when the only real outcome of concern is astrocytoma).

One way to evaluate confidence in a study is to examine how the authors present one’s own work. I had difficulty recognizing the descriptions of my own work. For example, a 2014 policy document I prepared for the Government of Australia followed a rigorous legal and policy framework for a presumption in the context of eligibility for compensation. This “framework” was clearly misunderstood by the authors, who thought it referred to an epidemiological framework, resulting in an egregious misrepresentation of my opinion on prostate cancer. The same report is classified as “descriptive” with methodology “unreported”, when, in fact, it was prepared for a specific purpose and for a specific client (Government of Australia), using specific criteria, to be read by senior civil servants and military officers. If authors wish to examine the grey literature, it behooves them to understand why and for whom it was written.

On the other hand, the authors ignored a much more recent, book-length treatise on these issues that described in copious detail the methodological issues (Guidotti TL. *Health Risk and Fair Compensation in the Fire Service*. Springer, 2015). Similarly, they relied upon my early 1995 interpretive paper and excluded my 2007 critique of methodology, which was of much more fundamental importance. (Citations are in the original paper.)

The literature on firefighting is cluttered with pretentious re-analyses that look at the same or overlapping datasets without understanding the problem at a deeper level. Progress will be made when we gain the understanding that precision in risk estimates is unattainable, accuracy is specific to populations, and that cancer risk among firefighters is, thankfully, a moving target. We also need to understand that standards of certainty are legitimately different for various applications of the findings: there is no one standard of scientific validity.

The authors are from a school of business, not health studies. They should be well aware that, under the law, in insurance business practice, and in compensation claims management, adjudication requires a level of proof that may be legitimately different from scientific certainty. Convergence of different lines of evidence is more important than consistency (precision) in risk estimates. Changes over the years may be much more revealing than treating firefighting as if it were frozen in time.

Superficially credible but deeply flawed publications such as this do harm to injured workers and confuse the compensation system, which is the most frequent and important user of this information.
